# The progression of lipid oxidation, β-carotenes degradation and sensory perception of batch-fried sliced sweet potato crisps during storage†

**DOI:** 10.1039/d0fo03100c

**Published:** 2021-04-19

**Authors:** Deepa Agarwal, Lim Mui, Emma Aldridge, James McKinney, Louise Hewson, Ian Denis Fisk

**Affiliations:** The New Zealand Institute of Plant and Food Research, Canterbury Agriculture & Science Centre Gerald St Lincoln 7608 New Zealand; School of Bioscience, University of Nottingham Sutton Bonington Loughborough LE12 5RD UK ian.fisk@nottingham.ac.uk; Pipers Crisps Ltd Pegasus Road Elsham Wold Brigg Lincolnshire DN20 0SQ UK; The University of Adelaide North Terrace Adelaide South Australia Australia

## Abstract

Dee are a unique and rapidly growing part of the global snack food market and are recognised as having distinct sensory properties (taste and texture). In this study, the development of important volatile aroma compounds over storage was evaluated and their chemical origin explained. Sweet potatoes were batch fried in high oleic sunflower oil (HOSO) and subjected to accelerated shelf life testing. Headspace volatiles were analysed using SPME GC-MS and correlated with sensory perception. All the components (sweet potatoes, oil and β-carotene) showed significant degradation after 3 weeks of storage at accelerated conditions (equivalent to 12 weeks in real-time at 25 °C). Marker volatiles associated with lipid oxidation such as hexanal, octanal, pentanal were identified, in addition to norisoprenoids from β-carotene degradation such as β-ionon, 5,6-epoxy-β-ionone, dihydroactinidiolide (DHA) and β-cyclocitral. The most prominent marker of lipid oxidation (hexanal) rapidly increased at week 1, whereas the carotene degradation makers did not rapidly increase until week 3 suggesting a delayed response. The frying temperature during the batch frying process of SPC was also shown to play a significant role in the sensory perception of the product over the shelf life. Overall, the results suggest that tight control of process variables and raw material design may enable extended shelf life and potentially enhanced health credentials for the product. These findings are unique to SPC, but also of value to the wider food industry.

## Abstract 2

Lipid oxidation markers, degradation markers of β-carotene and changes in sensory properties were tracked in fried sliced sweet potatoes over shelf life and under different frying conditions.

## Graphical abstract

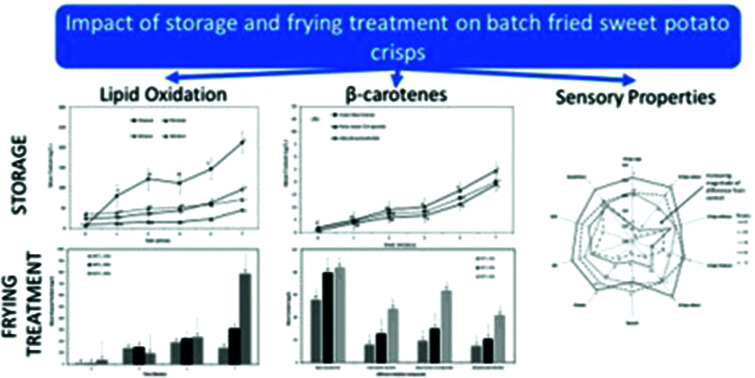

## 1. Introduction

Sweet potato (*Ipomomea batatas L*.) is a versatile dietary source of carbohydrates. Globally, sweet potato is the sixth most important food crop after rice, wheat, potatoes, maize and cassava. It is commonly consumed as mashed sweet potato, french-fries or it can be cooked by deep frying to form sliced sweet potato crisps (SPC) and then commonly salted. The frying process of sweet potatoes slices results in a flavour and texture which is unique and is well accepted by consumers.^1–5^ In a 2017 Mintel study, sweet potato was the highest rated alternative ingredient to standard potatoes in the crisp snack food category.^1^

Processing of sweet potatoes into crisps is much more challenging when compared to normal white potato crisps, this is due to major differences in the chemical composition and nutritional properties of the sweet potato, for example, sugar content, potential acrylamide formation and β-carotenes content.^6^ At the moment, food snack companies produce SPC either with higher moisture (which tends to compromise the shelf life of the product) or by mixing with other vegetable crisps such as carrots, beetroots *etc*. to control the overall nutritional content of the product. Alternatively, vacuum frying techniques are used to produce fried foods with low acrylamide content.^7^ Besides nutritional, there are significant differences in sensory attributes when comparing SPC and normal white potato crisps such as texture, taste and appearance. Hence, a fundamental understanding of the volatile aroma compounds of SPC and how it links to their sensory properties in relation to storage time and different processes is of critical importance.

There are number of parameters that influence the shelf life of deep-fried sweet potato crisps, such as lipid autoxidation and β-carotene degradation. Rancidity of edible oils due to lipid oxidation is a serious concern for all deep-fried or high fat-containing food products.^8,9^ Lipid hydroperoxides are the primary product of oxidation which are broken down to form aldehydes, ketones, alcohols, volatile organic acids, and epoxy compound productions, collectively these are considered secondary oxidation products.^10^ The presence of these compounds can result in a rancid off-taste, loss of nutritional value and reduced consumer acceptability. However, some of these lipid oxidation products (volatiles) are also responsible for the pleasant fried aroma of deep-fried snack foods which is important for the overall flavour profile, hence a good balance of these volatiles during production and maintaining these levels during storage is key. One route to manage the development of lipid oxidation is through the use of modified atmosphere packaging (MAP) to lower the oxygen concentration and thereby further supress development of lipid oxidation and loss of flavour quality during storage.^11^

Besides lipid oxidation, the degradation and loss of β-carotenes in SPC is also a critical parameter that determines the shelf life of the product. Degradation of carotenes has been widely studied in different food products such as carrots and sweet potatoes, the key factors that influence carotene oxidation are temperature, light, oxygen and acidity.^12,13^ Water activity is another important parameter for product quality, for example, it has been reported that higher level of β-carotene degradation was observed at lower water activities in freeze-dried SPC.^14^ Generally, in dried food products such as carrots or sweet potato crisps, β-carotene degradation showed a first-order kinetic reaction in the presence of one or more of these factors.^14,15^ The highly unsaturated structure of β-carotene reacts easily with radical species in a chain reaction, forming numerous secondary products. This is induced/accelerated by autoxidation (air), heating and enzymatic activity.^13,16,17^ The oxidised β-carotene degrades into epoxides, apocarotenals and apocarotenones, these, in later stages, oxidise into lower molecular weight carbonyl compounds, which are volatile, these include norisoprenoids.^18^ Norisoprenoids from autoxidation induced β-carotene degradation include β-ionon 5,6-epoxy-β-ionone, dihydroactinidiolide (DHA) and β-cyclocitral.^18,19^

Whilst there is a general understanding of the process of lipid oxidation and also β-carotene degradation, there is only a limited understanding of the simultaneous progression of both lipid oxidation and β-carotene degradation that occurs uniquely in deep batch-fried sliced SPC during storage. Therefore, we aim to evaluate and explain the volatile aroma compounds associated with the frying of deep batch fried SPC at different temperatures, and then correlate the development of these compounds with the sensory properties of the product over accelerated shelf life conditions. We aim to explain their chemical and biological origin specifically through β-carotene degradation and lipid oxidation. The findings are of use for industrial optimisation of the frying process of SPC and could be used to outline factors affecting the shelf life stability of SPC. The novelty of this approach lies in the fact that this fast method can be used to monitor both the progression of lipid oxidation and carotenoid degradation simultaneously in deep fried SPC.

## 2. Materials and methods

### 2.1. Materials

Three independent batches of “slightly salted” sweet potato crisps from same sweet potato variety were supplied from Pipers Crisps Ltd (Lincoln, UK), each batch was manufactured independently to ensure true replicates were used throughout. The basic composition of “slightly salted” SPC, fried at 140 °C for 150 s is 573 kcal per 100 g, carbohydrates 39.6% of which sugars are 18.4%, protein 4.6%, dietary fibre 7.6%, fat 45.2% (of which saturated 3.3%) and salt 0.51%, all data relates to a standard 100 g serving. 3-Heptanone used as an internal standard during SPME-GC-MS analysis was supplied by Sigma-Aldrich (UK).

### 2.2. Sweet potato crisps preparation

Freshly deep fried SPC were produced from a commercial batch frying unit (Pipers Crisps Ltd, UK). Three independent batches (30 kg) of unwashed sweet potato slices were batch fried in 100% high oleic sunflower oil (HOSO) (Kerfoot, Goole, UK) at three different temperatures (140 °C, 145 °C and 150 °C) and lightly seasoned with sea salt (1.0% w/w) (Helan Moon, UK) before packaging. Since there is no legal limit in the UK for FFA and TPM of frying oil, EU regulations suggest that for all deep-fried products when oil reaches 24% TPM, the frying life should be terminated and FFA should be maintained as low as possible typically below 0.5% due to the possible impact on the taste. Throughout the crisps frying process the free fatty acid (FFA) was maintained below 0.5% and total polar material (TPM) below 15% were maintained, to control the chemical quality of the frying oil.

#### 2.2.1. Accelerated shelf life sample preparation (ASLT)

In this study, ASLT was used as a tool to age the product at different temperatures and monitor the impact of storage with respect to temperature and time. There is no intent to define the shelf life of the product solely based on this methodology. All SPC samples were stored in closed chambers at 25 °C, 35 °C (data not shown) and 45 °C until further analysis. Bags were collected at fixed intervals (0, 1, 2, 3, 5 and 7 weeks) and stored individually in airtight containers at −80 °C prior to analysis. Reference samples were stored at −80 °C and used as a reference (week 0) for both SPME-GC-MS and sensory analysis.1
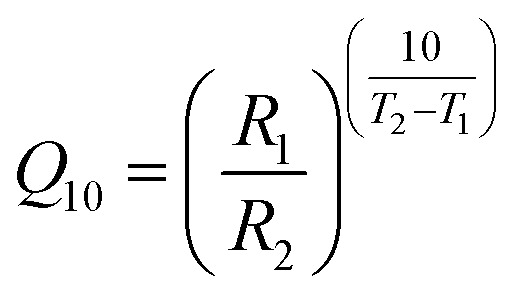


Autoxidation of the food products such as crisps is a relatively slow process at the ambient temperature, however, it can be accelerated by elevated temperature or relative humidity,^20,21^ and in the current study elevated temperatures were used for accelerated purposes. Accelerated shelf life testing can be expressed by using the *Q*_10_ concept. For the purpose of this study, an approximated *Q*_10_ value of 2 is used, meaning that the rate of oxidation doubled when storage temperature increased by 10 °C as an approximation. The aim of current investigation is not to work out the shelf life of the SPC, instead monitor the development of the volatile profile in correlation with sensory profile with storage time and frying profile in an industry relevant format.

#### 2.2.2. Headspace GC-MS sample preparation

Crisp samples were ground using a mortar and pestle (Fisherbrand™ Porcelain, UK). 2 g of crushed crisps sample were then mixed with 5 ml ultrapure water in 20 ml amber headspace vials (Supelco, Bellefonte, PA, USA) and 100 μl of 0.01% 3-heptanone in methanol (internal standard) was added. The vials were hermetically capped with PTFE-faced silicone septa (Supelco, Bellefonte, PA, USA).

### 2.3. Headspace SPME-GC-MS

The volatile aroma compounds associated with the sweet potato crisps were measured using gas phase solid phase micro-extraction (SPME) with GC-MS. The method was derived from ref. 11 as detailed below. The analysis was completed using an ISQ Single Quadrupole Mass Spectrometer, TRACE 1300 GC, with a TriPlus RSH autosampler (Thermo-Fisher Scientific, Waltham, MA, USA) and a ZB-WAX column (30 m × 0.25 mm I.D. × 1 μm film thickness). The SPME fibre was coated with a 50/30 μm layer of divinylbenzene–carboxen–polydimethylsiloxane (DVB/CARBOXEN/PDMS; Supelco) for analysis. Fibre exposure time in the headspace was 20 min at 70 °C. Subsequently, the fibre was thermally desorbed immediately for 4 min at 250 °C. The temperature ramp in the GC oven was: 40 °C (2 min), ramping to 240 °C at 6 °C min^−1^, hold at 240 °C for 5 min.^11^ MS was operated in electron impact (EI) ionisation mode at 70 eV and data acquisition was achieved at a scan rate of 0.20 s^−1^ over an *m*/*z* range of 35–300. The peak area was processed with Xcalibur Software and identification of aroma compounds using NIST library software (NIST/EPA/NIH Mass Spectral Library, version 2.0, Faircom Corporation, U.S.).

### 2.4. Sensory method

Sensory analysis was aimed to monitor the change in sensory perception of sweet potato crisps with increasing storage time. Full approval of the University of Nottingham local ethics committee was obtained before the study commenced. Informed consent was obtained from all assessors after the nature of the methods and nutritional consumption per session was fully explained.

Ten assessors (three male and seven female) was selected from the Pipers Crisps Ltd (UK) employee panel. All assessors were experienced in discrimination and descriptive tests and had previously worked extensively with SPC. Additionally, assessors were trained for minimum 50 h according to the ISO standard guidelines ISO 8586:2012 with focus on sensory method and product involved and including determination and training on key sensory attributes of the SPCs.^22^

The difference from control sensory test was identified as most suitable approach to evaluate if/when sensory differences occur over time and capturing the nature and magnitude of differences.^11,23^ All testing was carried out in one session, at an ambient temperature within a well-lit room. Each panellist was presented with the reference (fresh crisps produced on day 0, stored immediately at −80 °C) plus 5 samples (shelf-life samples stored up to 7 weeks) of SPC as described in section 2.2.1, each labelled with unique 3 digit codes. Panellists were instructed to compare each sample with the reference sample and for each sensory attribute indicate if a difference existed and the magnitude of difference. The magnitude of difference between reference and each sample was rated using a seven-point scale anchored as follows: 1 = very big difference, 2 = big difference, 3 = moderately big difference, 4 = moderate difference, 5 = slight difference, 6 = very slight difference and 7 = no difference. A range of sensory attributes were used to monitor the impact of ageing and frying conditions such as crisp appearance, crisp colour, crisp oiliness, flavour (salt) coating, flavour (salt) colour, crisp odour, crisp texture, potato taste, rancidity, salt perception and sweetness. Sensory panellists were encouraged to use the comments section to provide more specific information to elucidate the nature of any perceived differences in attributes. The order of presentation was randomised within a session and all panellists were required to cleanse their palate using the provided water (Evian, France) between the test samples and take a 5 min break after every 2 sets of samples.

### 2.5. Data analysis

All samples were prepared and analysed in triplicate. GC-MS data were analysed by XLSTAT 2009 (Addinsoft, USA), using analysis of variance with Tukey's *post hoc* test (*p*-value <0.05) to identify significant differences between samples sets. For the sensory data, the mean difference from control was calculated and subjected to analysis of variance (ANOVA), and when significant, followed by one tailed Dunnett's test for multiple comparisons with a control.

## 3. Results and discussion

**Fig. 1:**
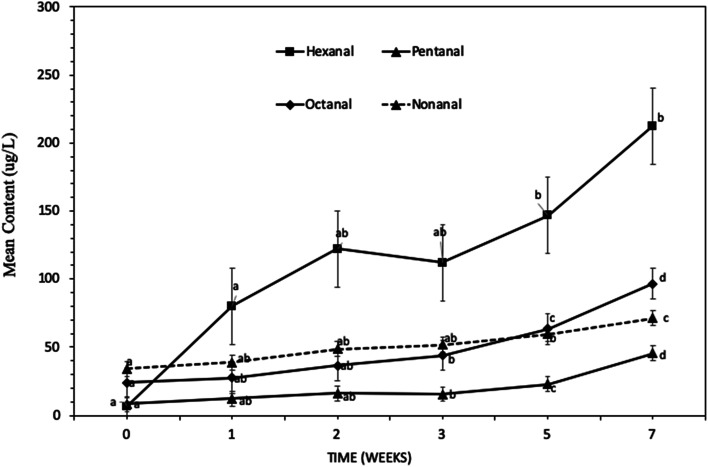
Change in some headspace volatiles compounds associated with lipid oxidation in deep-fried sweet potato crisps during storage (45 °C, for 7 weeks). Data is expressed as mean ± S.D. of 3 observations. ANOVA Tukey's *post hoc* test (*p*-value <0.05) was used for statistical analysis. Significant differences are represented by different letters.

A number of volatile compounds were observed in the headspace of deep-fried sweet potato crisps (SPC) during SPME-GC-MS analysis. These can be divided into 2 groups, *i.e.*, volatiles associated with lipid and sweet potato oxidation & β-carotene degradation with increasing storage time in sweet potato crisps. Volatile compounds associated with the autoxidation of lipids in deep fried SPC (at 145 °C for 150 s) are summarised in Table 1 with their sensory description. The most common secondary lipid oxidation markers were aldehydes such as hexanal, nonanal, heptanal, 2-pentyl furan, octanal, benzenacetaldehyde, benzaldehyde, pentanal, furfural, decanal, 2-decenal, 2,4-nonandienal, 2-undecenal, 2,4-decadienal, and alcohols such as 1-octanol and 1-heptanol.^11^ Similar lipid oxidation markers were observed for deep fried white potato crisps (WPC) and roasted peanuts.^28,30–32^ A number of studies have shown that hexanal is a good indicator for lipid oxidation in potato crisps and a typical undesirable off-flavour compound^10,33^ hence, the hexanal content was used to examine the progression of lipid oxidation over storage time. The hexanal content significantly increased (*p*-value <0.05) (Fig. 1) after 1 week of storage in accelerated conditions (1 week storage at 45 °C temperature is equivalent to 4 weeks in real-time at ambient temperature). Similarly, other volatile compounds associated with lipid oxidation such as pentanal, octanal and nonanal content showed a significant increase after 3 weeks storage.

**Table 1:** Headspace volatiles profile of the deep-fried sweet potato crisps by SPME-GC associated with lipid oxidation, sweet potatoes and β-carotene degradation with sensory description (sensory description is adapted from various literature)

Volatiles compounds^a^	Sensory description
Hexanal	Green, woody, grassy, rancid^10,24^
Heptanal	Fatty, green, oily^25^
2-Pentyl furan	Green, waxy, with musty^26^
Octanal	Oily, green, fatty, citrus^26^
Nonanal	Citrus, raw potato, oily, nutty^27^
1-Heptanol	Pungent, fruity, sweet, green^27^
Furfural	Brown, woody, nutty, caramellic with burnt astringent^27^
Decanal	Waxy, fatty, citrus and orange peel^27^
Benzaldehyde	Sweet, oily, almond, nutty^26^
1-Octanol	Waxy, green, citrus^27^
(*Z*)-2-Decenal	Waxy, fatty, earthy, green^27^
2,4-Nonandienal (*E*,*E*)-	Fatty, nutty, leaf^27^
2-Undecenal	Waxy, citrus peel^27^
2,4-Decadienal (*E*,*E*)-	Fatty, green, fried and potato^27^
3-Octen-2-one	Earthy, oily, sweet^27^
1-Octen-3-ol	Mushroom, earthy, fungal, green, oily^27^
Beta-cyclocitral	Tropical saffron, herbal, sweet tobacco, green, fruity^28^
*trans*-Beta-ionone	Floral, woody, violet^26^
Maltol	Sweet, caramellic, fruity, burnt^27^
2-Acetylpyrrole	Musty, nutty^27^
Beta-ionone-5,6-epoxide	Fruity, sweet, woody^29^
Dihydroactinidiolide	Ripe, apricot, red fruit, woody^29^

a Small traces of other volatiles were observed such as (*Z*)-2-heptanal, (*Z*)-2-octenal, benzeneacetaldehyde, *trans*-3-nonen-2-one, hexanoic acid, heptanoic acid, octanoic acid and nonanoic acid.

Other volatile compounds were observed and were specific to β-carotenes degradation in sweet potato (Table 1). Volatiles such as norisoprenoids *i.e.*, β-ionon 5,6-epoxy-β-ionone, dihydroactinidiolide (DHA) and β-cyclocitral are associated with the degradation of β-carotene in the sweet potatoes (similar volatiles were reported with dried sweet potato crisps and β-carotene).^34,35^ Volatile compounds associated with sugars degradation in sweet potatoes such as Maltol (sensory description: sweet, caramellic, fruity, burnt) and 2-acetylpyrrole (sensory description: musty, nutty) both showed a significant increase after 3 weeks storage (*p*-value <0.05).

**Fig. 2:**
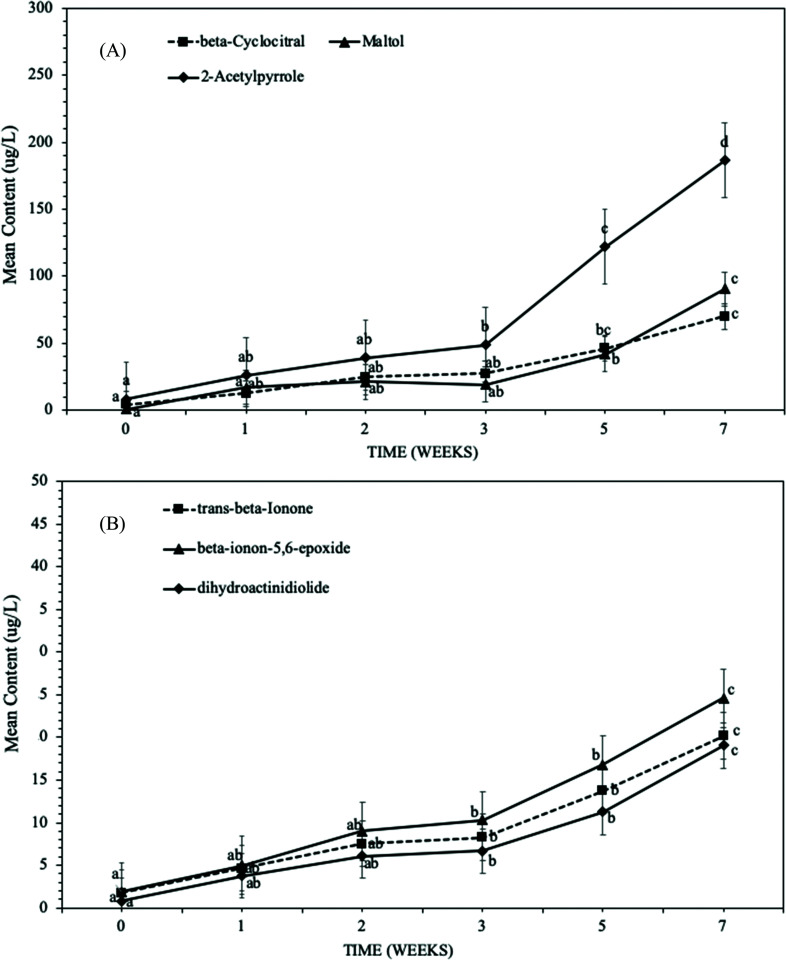
Change in headspace volatiles compounds associated with sweet potatoes in deep fried sweet potato crisps during storage at 45 °C for 7 weeks, where (a) beta-cyclocitral, Maltol and 2-acetylpyrrole, and (b) beta-ionon-5,6-epoxide, *trans*-beta-ionone and dihydroactinidiolide. Data is expressed as mean ± S.D. of 3 observations. ANOVA Tukey's *post hoc* test (*p*-value <0.05) was used for statistical analysis. Significant differences are represented by different letters.

Similarly, the volatiles associated with β-carotene degradation, *e.g.*, β-cyclocitral showed a significant increase after 3 weeks of storage (Fig. 2a), whereas volatiles such as β-ionon, 5,6-epoxy-β-ionone and dihydroactinidiolide (DHA), showed a significant increase (*p*-value <0.05) after 2 weeks (Fig. 2b). A similar dependency of volatiles associated with β-carotene degradation (storage temperature) and time was reported with dried SPC.^34^ Benchoff reported that 90% of the β-carotenes was degraded after 54 days of storage at 40 °C, whereas 35% when stored at 10 °C.^34^ In our studies, there was a significant increase in volatiles associated with β-carotene degradation after 3 weeks of storage at 45 °C, but our studies also highlight that some autoxidative products such as β-ionon, 5,6-epoxy-β-ionone and dihydroactinidiolide (DHA) are more sensitive compared to β-cyclocitral.

**Fig. 3:**
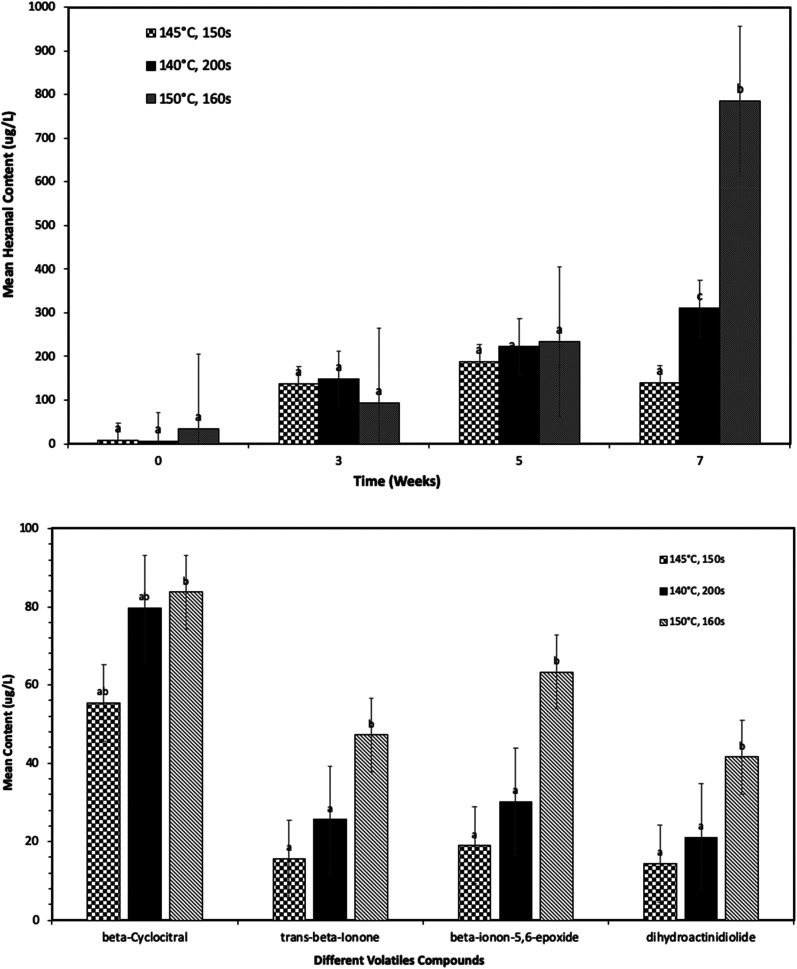
Impact of different frying conditions on (a) lipid oxidation (hexanal content), and (b) volatiles associated with sweet potatoes, over the shelf life of deep-fried sweet potato crisps after the storage at 45 °C for 7 weeks. Statistical significance is presented “abc”, in figure (a) comparing different frying profile at individual storage times, and (b) comparing different frying processes for individual volatiles after 7 weeks of storage. Data is expressed as mean ± S.D. of 3 observations. ANOVA Tukey's *post hoc* test (*p*-value <0.05) was used for statistical analysis. Significant differences are represented by different letters.

Fig. 3a and b shows the impact of different frying temperatures on the shelf life stability of the SPC. During this set of work unwashed slices of sweet potatoes were batch fried at different temperatures *i.e.*, 140 °C, 145 °C and 150 °C. The main rationale behind this set of work is to examine the impact of different cooking/frying temperatures on the volatiles associated with different components and sensory perception of the deep-fried SPC.

Fig. 3a shows the change in hexanal content of SPC fried at different temperatures as a function of storage time at 45 °C temperature. The hexanal content significantly increases (*p*-value <0.05) after 3 weeks of storage at 45 °C (data not shown), this is independent of the frying temperature, this behaviour was consistent with results presented in Fig. 1. However, a significant difference was observed with SPC fried at different temperatures after 7 weeks of storage at 45 °C (Fig. 3a), but no significant difference was observed up to 5 weeks of storage. For instance, crisps fried at a higher temperature 150 °C for 160 s had a significantly higher hexanal content (*p*-value <0.05) as compared to crisps fried at a lower temperature 145 °C for 150 s. Interestingly, crisps fried at a lower temperature *i.e.*, 140 °C for longer time 200 s, showed a significantly higher hexanal content (*p*-value <0.05) as compared to crisps fried for a shorter time. There are a number of factors that can impact oil update and stability such as slice thickness^36^ and pre-treatment of sweet potatoes slices (in present study sweet potatoes slices were unwashed) before frying may also affect the oil uptake at different frying temperature/time and subsequent lipid oxidation in the final product during storage. Besides that, the sweet potato cultivars may also affect the oil uptake, it is well reported that the variety of sweet potatoes has a significant influence on oil uptake in the deep-fried product,^30,31^ which is related to the cellular structures of different varieties affecting the moisture loss and subsequent oil uptake and total amount of volatiles associated with lipid oxidation in the product. However, further research is needed to determine the impact and mechanisms to confirm the impact of potato cultivar and cellular structures.

Volatile compounds such as β-ionon, 5,6-epoxy-β-ionone, dihydroactinidiolide (DHA) and β-cyclocitral associated with β-carotene degradation were significantly increased when comparing SPC fried at lower temperatures (Fig. 3b). It was evident in Fig. 3b, SPC when deep fried at higher temperature for shorter time *i.e.*, 150 °C for 160 s and at lower temperature for longer time *i.e.*, 140 °C for 200 s have higher rate of β-carotene degradation (an increased amount of volatiles contents) as compared to 145 °C. This significant increase in volatiles associated with β-carotenoid degradation was observed after 3 weeks of storage, which is consistent with the results presented in Fig. 2. The following results indicate that the frying temperature and frying time have a significant influence on the stability of the sweet potato crisps over shelf life. A similar impact of frying temperature on the shelf life stability of deep fried SPC and carrot chips was previously reported,^32,37^ where deep-frying at different temperatures led to a significant difference in oil content, moisture content and sensory perception of the product.

Acrylamide formation is one of the challenges with deep fried products such as normal white potato crisps and SPC. It is well established that the frying at low temperature and pre-treatment significantly reduced the acrylamide formation in deep-fried products.^33^ When comparing the acrylamide formation with different frying temperature presented in this study, the acrylamide formation was higher with sweet potato crisps fried at higher temperature (150 °C) as compared to crisps fried at lower temperature (145 °C) (data shown in ESI†) and these results were consistent with published data.^33^

**Fig. 4:**
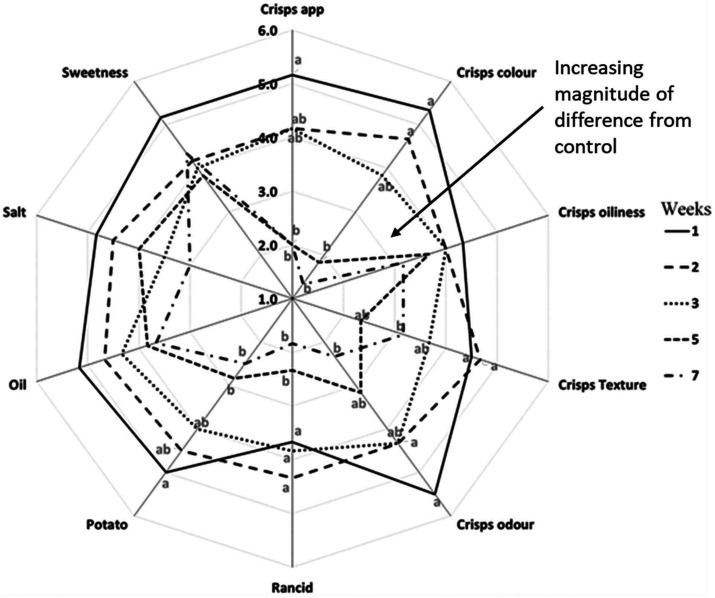
Impact of ageing on the sensory properties of deep-fried sweet potato crisps during storage at 45 °C for 7 weeks. The magnitude of difference of the samples was rated against a control/reference sample using a seven-point category scale anchored as follows: 1 = very big difference, 2 = big difference, 3 = moderately big difference, 4 = moderate difference, 5 = slight difference, 6 = very slight difference and 7 = no difference. One-tailed Dunnett's test for multiple comparisons with a control was used for statistical analysis (*p*-value <0.05). Significant differences are represented by different letters.

There was a significant change in the sensory perception of SPC over accelerated shelf life testing (Fig. 4). Sensory perception of aged SPC showed a significant step-change after 3 weeks of storage (Fig. 4). The sensory attributes such as crisp appearance; crisp colour; texture and crisp odour significantly changed (*p*-value <0.05) with increasing storage time. Both the appearance of the crisps and their colour significantly changed, compared to reference, after 3 weeks of storage at 45 °C storage temperature (*p*-value <0.05), with panellists reporting changes in colour from orange to dark brown. The change in appearance and colour in Fig. 4 can be explained by the storage temperature as the effect observed at 45 °C wasn't reported for samples stored at a 20 °C (data not shown). Similar behaviour was reported with carrot chips and ground red pepper (both products also have a high carotene content), where an increase in storage temperature led to an increase in the rate of both α- & β-carotene degradation, resulting in a change in colour.^38,39^ This is of industrial relevance when developing new product for export to markets with higher atmospheric temperatures. Besides the appearance, texture and odour of the SPC were significantly affected by storage time (*p*-value <0.05). Similarly, a significant change (*p*-value <0.05) in crisp odour (stale odour) and rancid perception was reported after 3 weeks of storage (Fig. 4). The following change in rancid/stale can be explained by the significant increase in volatiles associated with lipid oxidation at 3 weeks, initially starting with hexanal in week 1 (Fig. 1). Whereas the off-taste such as burnt/bitter in SPC can be explained by an increase in the volatiles associated with sweet potato and β-carotene degradation observed in Fig. 2a and b.

There were no significant changes to saltiness, which is to be expected as sodium chloride will not degrade over the temperature range experienced in the shelf life study. It is interesting to note that salt historically has been added to mask off-notes in high fat products that are likely to become rancid over storage, and therefore salt may serve a similar role in salted snack products. A slower development of oxidative rancidity and β-carotene degradation through tight control of process variables and raw material design may therefore enable a sodium reduction in the product as less salt is required to mask off notes, and when combined with a reduced acrylamide formation, could ultimately result in enhanced health credentials for the products.

Future work could include correlating the chemical changes with the physical properties of the SPC, specifically image analysis/colour may be of benefit and could be used within the food industry as a rapid test for commercial applications. Additional compositional analysis of beta-carotene, hydroperoxide formation and the degradation of fatty acid over shelf life would be of use in future studies to further help correlate the volatile markers with the chemical state of the product.

## 4. Conclusion

Sweet potato crisps are relatively more sensitive to deep frying as compared to normal white potato crisps. A wide range of volatiles associated with lipid oxidation, β-carotene degradation and sweet potatoes were identified in deep fried and lightly salted sweet potato crisps. A significant increase in volatiles associated with lipid, sweet potatoes and β-carotene degradation was evident after 3 weeks of storage at a 45 °C temperature, whereas an increase in hexanal (an early marker of lipid oxidation) was apparent from week 1. Higher frying temperatures increased the development of volatiles associated with lipid oxidation and β-carotene degradation and development of greater changes in texture, and burnt and bitter sensory properties. In all, the findings suggest that tight control of process variables and raw material design may enable extended shelf life and potentially enhanced health credentials for this unique product.

## Author contribution

All authors have made substantial contributions to conception and design of the project. All authors have critically revised and approved the final submitted version of the manuscript. Experimental work was carried out by DA.

## Ethical statements

All experiments were performed in accordance with the University of Nottingham Quality Manual. Experiments were approved by the local ethics committee at the University of Nottingham. Informed consent was obtained from human participants of this study after the nature of the methods and nutritional consumption per session was fully explained.

## Conflicts of interest

There are no conflicts of interest to declare.

## Supplementary Material

FO-012-D0FO03100C-s001
