# Supplementary material for: The progression of lipid oxidation, β-carotenes degradation and sensory perception of batch-fried sliced sweet potato crisps during storage
Source: Food Funct. 2021 Apr 19;12(10):4535–43. doi: 10.1039/d0fo03100c (PMC8145155; doi:10.1039/d0fo03100c)
Supplement: FO-012-D0FO03100C-s001 [file FO-012-D0FO03100C-s001.pdf]

Electronic Supplementary Material (ESI) for Food & Function.
This journal is © The Royal Society of Chemistry 2021

Supporting information

Supplemental Figures

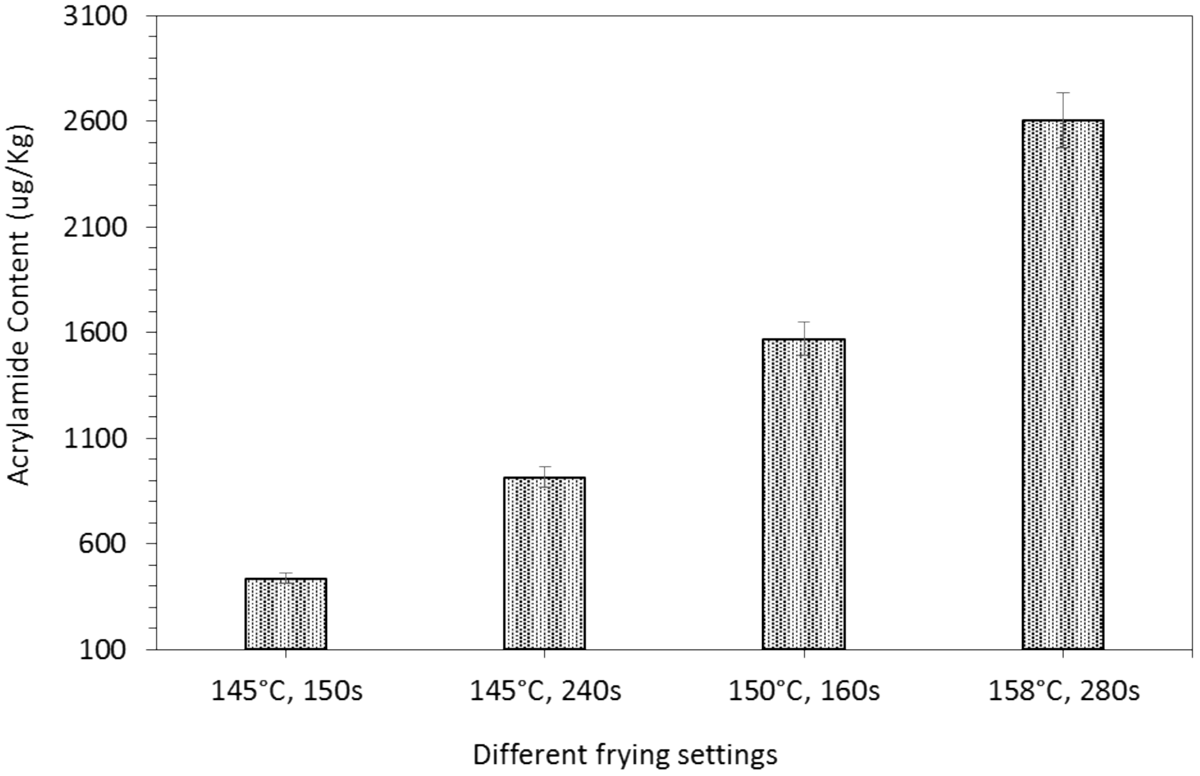

Figure S1: *Acrylamide content in sweet potato crisps fried in HOSO at different frying settings.*
